# Targeted Therapies in Advanced Cholangiocarcinoma

**DOI:** 10.3390/life13102066

**Published:** 2023-10-16

**Authors:** Michael H. Storandt, Peter C. Kurniali, Amit Mahipal, Zhaohui Jin

**Affiliations:** 1 Department of Internal Medicine, Mayo Clinic, Rochester, MN 55905, USA; storandt.michael@mayo.edu; 2 Department of Hematology/Medical Oncology, Sanford Cancer Center, Bismarck, ND 58501, USA; peter.kurniali@sanfordhealth.org; 3 Department of Internal Medicine, Division of Hematology/Oncology, University of North Dakota School of Medicine and Health Sciences, Grand Forks, ND 58203, USA; 4 Department of Oncology, University Hospitals Seidman Cancer Center and Case Western Reserve University, Cleveland, OH 44106, USA; amit.mahipal@uhhospitals.org; 5 Department of Medical Oncology, Mayo Clinic, Rochester, MN 55905, USA

**Keywords:** cholangiocarcinoma, targeted therapy, systemic therapy

## Abstract

Primary tumor resection and liver transplantation are the only curative treatment options for the management of cholangiocarcinoma (CCA). However, for patients with advanced or metastatic disease, palliative systemic therapy remains the only treatment option. The development of targeted therapeutics has begun to shift the treatment paradigm in CCA. Targets of interest in CCA include mutated isocitrate dehydrogenase-1 (mIDH-1), human epidermal growth factor receptor 2 (HER2) overexpression/amplification, and fibroblast growth factor receptor 2 (*FGFR2*) fusion, in addition to less frequently observed targets such as *BRAF V600E*, deficient mismatch repair/high microsatellite instability (dMMR/MSI-H), and high tumor mutation burden (TMB-H). These targets are observed in varying frequency among patients with intrahepatic CCA and extrahepatic CCA. Multiple novel therapies have been developed to exploit each of these targets, with some having received United States Food and Drug Administration approval for use in the second-line setting. In the current review, we discuss targets of interest in CCA and summarize current evidence evaluating available therapies directed at these targets.

## 1. Introduction

Cholangiocarcinoma (CCA) is a malignancy of the biliary epithelium that accounts for approximately 3% of gastrointestinal malignancies globally [1]. CCA is subclassified based on its anatomic location and may be categorized as intrahepatic (iCCA), which accounts for 10–20% of CCA, or extrahepatic, which accounts for 80–90% of CCA in the United States (U.S.) [2,3]. CCA exhibits geographic trends in incidence, with the highest rates observed in Southeast Asia, and substantially lower rates in Western nations [4,5]. This is related to its unique risk factor profile, with risk factors including liver flukes, hepatolithiasis, and hepatitis B virus in Asian countries, and more commonly cholangitis in the U.S. [1,5]. Of note, a recent study has suggested an increase in rates of CCA in the United States, with this increase primarily seen in iCCA [6,7,8,9]. Surgical resection or transplant in select cases are the only curative options for CCA, and cases advanced beyond surgical resection are managed with palliative systemic therapy and, in select cases, liver-directed therapy.

Previously, standard first-line systemic therapy for advanced CCA consisted of gemcitabine plus cisplatin based on the ABC-02 trial that demonstrated survival benefit with the combination regimen compared to gemcitabine monotherapy, and this remained the standard of care for over a decade [10]. More recently, a phase 2 trial evaluating gemcitabine plus cisplatin with the addition of durvalumab, a monoclonal antibody targeting programmed death-ligand 1 (PD-L1), demonstrated an objective response in 34 of 47 patients (70%) [11]. This was followed by TOPAZ-1, which is an ongoing global phase 3 trial enrolling patients with advanced, previously untreated biliary tract cancers, in which patients were randomized to receive gemcitabine plus cisplatin with or without durvalumab [6]. Interim analysis of this study found that the addition of durvalumab to chemotherapy led to improved median progression-free survival (mPFS) [7.2 vs. 5.7 months, hazard ratio (HR) 0.75, 95% confidence interval (CI) 0.64–0.89, *p* = 0.001] and median overall survival (mOS) (12.8 vs. 11.5 months, HR 0.80, 95% CI 0.66–0.97, *p* = 0.021) [12]. The TOPAZ-1 trial established gemcitabine plus cisplatin with durvalumab as standard first-line systemic therapy for advanced CCA. 

More recently, KEYNOTE-966, which was a randomized, double-blind, placebo-controlled, phase 3 trial, evaluated patients with advanced, previously untreated biliary tract cancers who were randomized to receive gemcitabine plus cisplatin with pembrolizumab, an anti-programmed cell death-1 (anti-PD-1) antibody, or placebo [13]. Patients receiving chemotherapy plus pembrolizumab manifested an improved mOS of 12.7 months versus 10.9 months in the placebo arm (HR 0.83, 95% CI 0.72–0.95, *p* = 0.0034). This regimen has not yet received U.S. Food and Drug Administration (FDA) approval but represents an alternative first-line option for advanced CCA. 

Following progression on first-line systemic therapy, second-line therapy may consist of folinic acid, fluorouracil, and oxaliplatin (FOLFOX), after the ABC-06 trial, which was an open-label, randomized, phase 3 trial consisting of patients with advanced biliary tract cancer who had progression on first-line gemcitabine plus cisplatin and were randomized to active symptom control with or without FOLFOX, found that patients in the FOLFOX arm exhibited longer mOS (6.2 months vs. 5.3 months, HR 0.69, 95% CI 0.50–0.97, *p* = 0.031) [14]. Alternatively, NIFTY, a phase 2b, open-label, randomized trial conducted in South Korea, randomized patients with metastatic biliary tract cancer who had progressed on first-line gemcitabine plus cisplatin to fluorouracil plus leucovorin with or without liposomal irinotecan [15]. The irinotecan-containing arm was able to achieve its primary endpoint of improved mPFS (4.2 vs. 1.7 months, HR 0.61, 95% CI 0.44–0.86, *p* = 0.004) [16]. However, NALIRICC-AIO-HEP-0116, a phase 2 trial conducted in Germany in which patients were randomized to receive fluorouracil plus leucovorin with or without liposomal irinotecan, failed to demonstrate significant improvement in mPFS (2.8 vs. 2.3 months) or mOS (6.9 vs. 8.2 months), and the liposomal irinotecan-containing regimen was associated with greater toxicity [17].The difference in outcomes between these two trials may be explained by multiple differences. NIFTY was conducted in an Asian population, among which 42.5% had intrahepatic disease, while NALIRICC-AIO-HEP-0116 was conducted in a European population, among which 64% had intrahepatic disease. Additionally, the liposomal irinotecan dose was 70 mg/m^2^ in NIFTY but was 80 mg/m^2^ in NALIRICC-AIO-HEP-0116. At this time, fluoropyrimidine plus irinotecan may be considered a second- or third-line option in CCA, but the efficacy of irinotecan-based regimens is not well established. Select trials establishing first- and second-line standards of care are listed in Table 1.

**Figure 1:**
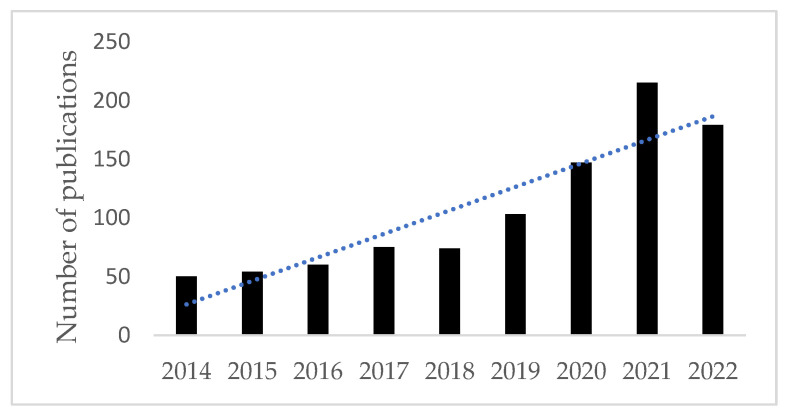
Number of publications indexed on PubMed per year using search terms “targeted” and “cholangiocarcinoma”.

Given the marginal difference in response rate and overall survival with different combinations of cytotoxic chemotherapy and immunotherapy in advanced CCA and the detection of actionable targets in nearly 50% of CCA cases, greater interest has been generated in targeted therapy for the management of CCA [18]. This has subsequently led to the development of novel therapeutics, altering the treatment paradigm for CCA [19,20]. Figure 1 depicts an increase in publications using the search terms “targeted” and “cholangiocarcinoma” over the past 10 years. The following article will review current targets of interest in advanced CCA and discuss current trial data evaluating the efficacy of novel therapies directed at these targets.

## 2. IDH-1 Mutation

### 2.1. IDH-1 Mutation Pathophysiology

Isocitrate dehydrogenase-1 (IDH-1) is a metabolic enzyme that functions to convert isocitrate to α-ketoglutarate, and mutations of this enzyme have been implicated in oncogenesis [21]. Specifically, the *IDH-1* mutation (mIDH-1) may result in a gain of function, facilitating the conversion of α-ketoglutarate to D-2-hydroxyglutarate, which is an oncometabolite [21,22]. Accumulation of D-2-hydroxyglutarate is thought to result in epigenetic dysregulation, resulting in impairment of cellular differentiation [21]. Mutation of *IDH-1* has been implicated in glioma and acute myeloid leukemia, as well as iCCA, where it may be detected in 13–25% of cases [23,24,25]. Detection in eCCA is rare and may be present in <1% of patients [25]. It is unclear if mIDH-1 is associated with a difference in overall survival and progression-free survival when compared to CCA patients lacking this mutation [25,26].

### 2.2. IDH-1 Targeted Therapy

Ivosidenib is a targeted inhibitor of mIDH-1 and was first used in the management of mIDH-1 acute myeloid leukemia [27]. It was first evaluated for use in solid malignancies in a phase 1 dose escalation and expansion study enrolling patients with an mIDH-1 solid malignancy that had progressed on at least one prior line of therapy [28]. This study included 73 patients with mIDH-1 CCA. Among patients with CCA, the most common treatment-related toxicities included fatigue (42%), nausea (34%), diarrhea (32%), abdominal pain (27%), decreased appetite (27%), and vomiting (23%). Grade 3 or worse toxicities included ascites (5%), anemia (4%), and fatigue (3%). Overall, 5% of the total study population had a partial response, and mPFS was 3.8 months (95% CI 3.6–7.3).

This trial was subsequently followed by ClarIDHy, which was a multicenter, randomized, double-blind, placebo-controlled, phase 3 trial evaluating ivosidenib in 230 patients with mIDH-1 CCA who had progressed on at least one prior line of therapy [29]. Patients were randomized 2:1 to receive ivosidenib versus placebo, and, of note, patients were allowed to crossover to the ivosidenib arm at disease progression. Only 3 patients (2%) in the ivosidenib arm had a partial response; however, 51% had stable disease, which is consistent with the known mechanism of ivosidenib, which is not a cytoxic drug but inhibits mIDH-1, allowing for cellular differentiation. This study ultimately achieved its primary endpoint of improved mPFS, primarily driven by a high rate of stable disease, with those receiving ivosidenib manifesting an mPFS of 2.7 months versus 1.4 months in the placebo arm (HR 0.37, 95% CI 0.25–0.54, *p* < 0.0001). Final survival analysis failed to demonstrate a statistically significant improvement in survival, with an mOS of 10.3 months in the ivosidenib arm versus 7.5 months in the placebo arm (HR 0.79, 95% CI 0.56–1.12, *p* = 0.09) [30]. However, the survival benefit may be diluted by the fact that crossover was allowed in this trial, and 70% of patients in the placebo arm received ivosidenib after progression. Rank-preserving structural failure time-adjusted mOS analysis accounting for crossover did demonstrate improved overall survival (HR 0.46, 95% CI 0.28–0.75, *p* = 0.0008). In addition to the above-mentioned benefits, those receiving ivosidenib did not experience a decline in quality of life when compared to placebo. Based on the results of ClarIDHy, the U.S. FDA granted approval of ivosidenib for patients with advanced mIDH-1 CCA who had progressed on first-line therapy.

## 3. HER2 Overexpression and/or Amplification

### 3.1. HER2 Overexpression and/or Amplification Pathophysiology

Human epidermal growth factor receptor 2 (HER2) is a receptor tyrosine kinase that, when activated, results in activation of the PI3K-AKT-mTOR pathway [31,32]. Overexpression of HER2 may occur through HER2 gene amplification, which may in turn result in the formation of homodimers or heterodimers with other HER proteins, such as HER3, leading to activation of downstream signaling pathways [32,33]. HER2 amplification has been implicated in multiple malignancies, most commonly bladder cancer, followed by esophageal/gastroesophageal junction, breast, gallbladder, eCCA, gastric, and cervical cancer [34]. HER-2 amplification/overexpression may be detected in approximately 17% of cases of eCCA and 5% of cases of iCCA [35]. HER2 amplification/overexpression has been associated with shorter disease-free survival following curative resection of biliary tract malignancies [36]. Evidence is limited with regards to the prognostic implications of HER2 amplification/overexpression in advanced CCA, but some evidence may suggest that it does not impact patient prognosis significantly [37].

### 3.2. HER2 Overexpression/Amplification Targeted Therapy

Trastuzumab is a monoclonal antibody that binds domain IV of HER2, resulting in antibody-dependent cell-mediated cytotoxicity, while pertuzumab, which has shown synergism when used with trastuzumab, is a monoclonal antibody that binds subdomain II of HER2, blocking dimerization and therefore activation of HER2 [38]. HER2-directed therapy in CCA has been evaluated in MyPathway, which was a non-randomized, multicenter, open-label, phase 2a basket study that enrolled 39 patients with advanced, previously treated biliary tract malignancies with HER2 amplification and/or overexpression [39]. Patients enrolled would receive pertuzumab plus trastuzumab with a primary endpoint of objective response rate (ORR). ORR was 23%, with 23% of patients having a partial response. Median PFS was 2.6 months (95% CI 1.0–5.3) for seven patients with iCCA and 6.8 months (95% CI 1.3–13.5) for 7 patients with eCCA. Patients with iCCA manifested a mOS of 3.9 months (95% CI 1.2–8.1), while those with eCCA manifested a mOS of 8.0 months (95% CI 2.0–not estimable). Ninety-two percent of patients developed treatment-emergent adverse events (TEAEs), with the most common grade 1–2 events including diarrhea (33%), alanine aminotransferase (ALT) elevation (18%), aspartate aminotransferase (AST) elevation (18%), fatigue (18%), anemia (15%), pyrexia (15%), and nausea (15%). Forty-six percent of patients had a grade 3–4 TEAE, with the most common including ALT elevation (13%), AST elevation (13%), and alkaline phosphatase elevation (10%). Based on the results of this trial, pertuzumab plus trastuzumab may be considered a second-line option for HER2 overexpressing/amplified CCA.

HERIZON-BTC-01 was a global, phase 2b trial evaluating zanidatimab, a monoclonal antibody binding HER2 at two domains, resulting in the formation of HER2-antibody clusters that are internalized, reducing HER2 expression, in patients with advanced, HER2-amplified biliary tract cancers with progression on gemcitabine-based therapy [40]. Among 80 patients with HER2 2+/3+ expression, an ORR of 41.3% was observed with an mPFS of 5.5 months. Fifty-four percent experienced a grade 1 or 2 treatment-related adverse event (TRAE), the most common of which included diarrhea, infusion-related reactions (32% each), and nausea (8%). Eighteen percent had a grade 3 or greater adverse event, including diarrhea (5%), ejection fraction decrease (3%), and anemia (2%). This study demonstrates the overall tolerability of zanidatimab, with promise for use in HER2-positive biliary tract malignancies.

DESTINY-PanTumor02 was an open-label, phase 2 trial evaluating trastuzumab deruxtecan, a topoisomerase inhibitor conjugated to trastuzumab, in patients with HER2-positive, previously treated solid malignancies, excluding breast, gastric, colorectal, and non-small-cell lung cancer [41]. Among the 267 patients enrolled, an ORR of 37.1% was seen with a median duration of response (mDOR) of 11.8 months. This study included 41 patients with biliary tract cancer who had an ORR of 22.0%. When looking at 16 patients with biliary tract cancer with HER2 3+ expression by IHC, an ORR of 56.3% was seen. Among all patients, grade 3 or greater adverse events were reported in 58.4%, with 11.6% discontinuing treatment due to adverse events. Overall, this study suggests a role for trastuzumab deruxtecan in HER2-positive CCA, primarily those with 3+ expression, based on the high response rate seen in this trial.

## 4. FGFR2 Fusion or Rearrangement

### 4.1. FGFR2 Fusion or Rearrangement Pathophysiology

Fibroblast growth factor receptor 2 (FGFR2) is a member of a family of four receptor tyrosine kinases that, when activated by fibroblast growth factors, dimerize, leading to autophosphorylation of the intracellular kinase domain and activation of downstream signaling pathways [42,43,44,45]. Dysregulation of FGFR2 may play a role in oncogenesis through angiogenesis and the migration and survival of tumor cells [46,47]. *FGFR2* fusion seen in CCA is most commonly due to chromosomal rearrangement causing a C-terminal substitution of *FGFR2* to the region of a binding partner, allowing auto-dimerization of FGFR2 and constitutive activation of downstream signaling pathways [48,49]. *FGFR2* fusion is primarily seen in iCCA and rarely other cancers, including gallbladder carcinoma, hepatocellular carcinoma, pancreatic cancer, and sarcoma. In iCCA, frequency was initially reported in approximately 15% of cases, but in advanced iCCA, the frequency may be lower in 7–10% [50,51]. *FGFR2* alterations have been associated with improved survival in iCCA, even without the use of FGFR inhibitors [52,53].

### 4.2. FGFR2 Fusion or Rearrangement Targeted Therapy

*FGFR2* is one of the best studied targets with regard to therapeutic options in CCA, and multiple FGFR inhibitors have received FDA approval for the indication of CCA, including pemigatinib, futibatinib, and infigratinib [49].

#### 4.2.1. Pemigatinib

Pemigatinib is a selective inhibitor of FGFR1-3 and was the first FGFR inhibitor to receive FDA approval for treatment of CCA [54,55]. It was first evaluated in the phase 1/2 dose escalation/expansion trial, FIGHT-101, which ultimately determined a dose of 13.5 mg for the expansion portion of the trial [56]. This study included 21 patients with CCA, of whom 5 exhibited a partial response. The most common TEAEs included hyperphosphatemia (75.0%), fatigue (39.1%), dry mouth (38.3%), stomatitis (34.4%), diarrhea (32%), and alopecia (31.3%). The most common grade 3 or greater toxicities included fatigue (10.2%), stomatitis (8.6%), and anemia (7.0%). Clinically notable TEAEs included hyperphosphatemia (75%), nail toxicity (32%), dry eye (23.4%), blurred vision (16.4%), hypophosphatemia (15.6%), and eyelash changes (13.3%).

This was followed by FIGHT-202, which was a multicenter, open-label, phase 2 trial enrolling 3 cohorts of patients with CCA who had progressed on prior treatment: those with *FGFR2* fusion or rearrangement, those with *FGFR2* mutation, and those with no alteration of *FGFR2* [57]. Among patients with *FGFR2* fusion or rearrangement, 35.5% achieved an objective response, demonstrating clinical promise. Patients with *FGFR2* fusion or rearrangement demonstrated an mPFS of 6.9 months (95% CI 6.2–9.6) and an mOS of 21.1 months (95% CI 14.8–not estimable). An additional phase 2 study evaluating pemigatinib in Chinese patients with advanced CCA who had progressed on prior therapy found an ORR of 50.0% [58]. Based on the results of FIGHT-202, pemigatinib was granted FDA approval for treatment of CCA with *FGR2* fusion or rearrangement following progression on first-line therapy.

FIGHT-302 is currently ongoing, which is an open-label, randomized, active-control, phase 3 study evaluating first-line pemigatinib versus gemcitabine plus cisplatin in the treatment of CCA with *FGFR2* rearrangement [59]. This study has the potential to impact the first-line treatment of CCA with *FGFR2* fusion or rearrangement.

#### 4.2.2. Futibatinib

Futibatinib is an irreversible, pan-FGFR inhibitor [60]. It was first evaluated in a phase 1 trial enrolling 86 patients with a history of advanced solid malignancy with progression on prior therapy, among whom 83% harbored an FGF/FGFR aberration [61]. This trial included 24 patients with CCA, and of the 5 patients who had a partial response, 3 were among those with CCA known to harbor an *FGFR* abnormality. The most frequently observed TEAEs included hyperphosphatemia (59%), diarrhea (37%), and constipation (34%), with 48% of patients experiencing grade 3 toxicity. Futibatinib was also evaluated in an additional phase 1 trial including 197 patients with advanced solid tumors, with an ORR of 25.4% in patients with *FGFR2* fusion/rearrangement-positive iCCA [62].

These were followed by FOENIX-CCA2, which was a multinational, open-label, phase 2 study including 103 patients with advanced iCCA with *FGFR2* fusion/rearrangement who had progressed on prior therapy [63]. An ORR of 52% was observed, and mPFS was 9.0 months (95% CI 6.9–13.1) and mOS was 21.7 months (95% CI 14.5-not evaluable). Later, updated analysis reported an ORR of 41.7%, mPFS of 8.9 months, and mOS of 20.0 months [64]. Based on the results of FOENIX-CCA2, futibatinib received FDA approval for advanced, *FGFR2* fusion/rearrangement-positive iCCA with progression on prior therapy. FOENIX-CCA3 was a planned phase 3 trial to evaluate futibatinib versus gemcitabine plus cisplatin in the first-line setting; however, this trial has been halted due to slow patient accrual [65].

#### 4.2.3. Infigratinib

Infigratinib is a pan-FGFR inhibitor that was first evaluated in a phase 1 dose escalation/expansion study that included 132 patients with solid malignancies with *FGFR* alterations [66,67]. This study determined a maximum tolerated dose of 125 mg based on dose-limiting toxicities.

Infigratinib was subsequently evaluated in an open-label, phase 2 trial that enrolled patients with advanced CCA harboring an *FGFR2* fusion or alteration who had progressed on prior therapy [68]. An ORR of 14.8% and a disease control rate (DCR) of 75.4% were observed, and among 48 patients with an *FGFR2* fusion, an ORR of 18.8% and a DCR of 83.3% were observed. The most commonly observed TEAEs included hyperphosphatemia (72.1%), fatigue (36.1%), stomatitis (29.5%), and alopecia (26.2%), with grade 3 or 4 TEAEs in 41%, including hyperphosphatemia (16.4%), stomatitis (6.6%), and palmar-plantar erythrodysesthesia (4.9%). An additional open-label, phase 2 study enrolling 122 patients with advanced CCA with *FGFR2* fusion or rearrangement with progression on prior gemcitabine-based chemotherapy found an ORR of 23.1% (95% CI 15.6–32.2) [69]. Based on the results of these phase 2 trials, the FDA has granted approval for the use of infigratinib for previously treated, advanced CCA harboring an *FGFR2* fusion or rearrangement. Despite this, its manufacturer will no longer pursue this clinical indication for infigratinib, and further trials have been discontinued.

## 5. BRAF V600E

### 5.1. BRAF V600E Pathophysiology

*BRAF* is a proto-oncogene *that* functions within the mitogen-activated protein kinase/extracellular signal-related kinase (MAPK/ERK) pathway and plays a role in cell proliferation [70]. *BRAF V600E* is the most common mutation of *BRAF*, resulting in constitutive activation of this signaling pathway. *BRAF V600E* has been implicated in multiple malignancies, including melanoma, colorectal cancer, hairy cell leukemia, and multiple myeloma [70]. *BRAF V600E* has been detected in 1–5% of cases of CCA and is primarily found in iCCA [71,72,73,74,75,76]. The presence of *BRAF V600E* has prognostic implications in iCCA and is associated with shorter overall survival and disease-free survival [71]. Additionally, when comparing patients with *BRAF V600E* to patients with *non-V600E BRAF* mutations, patients with *BRAF V600E* are more likely to have larger tumors, more synchronous tumors, and increased invasion of vessels or bile ducts [71].

### 5.2. BRAF V600E Targeted Therapy

Dabrafenib, a selective BRAF inhibitor, and trametinib, a MEK1/2 inhibitor, were evaluated via subprotocol H (EAY131-H) of the NCI-MATCH platform trial, which was an open-label, single-arm study enrolling 35 patients with a *BRAF V600E* mutated solid malignancy, excluding patients with melanoma, thyroid, colorectal, and non-small-cell lung cancer [77]. This study demonstrated an ORR of 38%, and among 4 patients with CCA, 3 exhibited a partial response. The most common TEAEs included fatigue (74%), nausea (57%), fever/chills (51 and 54% respectively), headache (29%), alkaline phosphatase elevation (31%), and AST elevation (29%). The most common grade 3 TEAEs included fatigue (13%), neutrophil count decrease (8.6%), white blood cell count decrease (8.6%), and hyponatremia (5.7%).

Dabrafenib plus trametinib was subsequently evaluated in ROAR, which was an open-label, single-arm, phase 2 basket study that included a biliary tract cancer cohort consisting of 43 patients with *BRAF V600E*-mutated, advanced biliary tract cancer with progression on prior therapy [78]. The ORR was 51% with an mPFS of 9 months (95% CI 5–10) and mOS of 14 months (95% CI 10–33). Dabrafenib and trametinib subsequently received FDA approval for advanced *BRAF V600E*-mutated solid malignancies with progression on prior therapy.

## 6. Deficient Mismatch Repair/High Microsatellite Instability

### 6.1. Deficient Mismatch Repair/High Microsatellite Instability Pathophysiology

Mismatch repair proteins, including MSH2, MSH6, MLH1, and PMS2, function to correct errors that occur during DNA replication [79,80]. Loss of function of one or more of these proteins is referred to as deficient mismatch repair (dMMR), which results in high microsatellite instability (MSI-H) through the accumulation of mutations within the cell. Deficient mismatch repair may occur sporadically or through germline mutation, which is referred to as Lynch syndrome. Deficient mismatch repair may be gauged by immunohistochemistry evaluation of the expression of mismatch repair proteins or through polymerase chain reaction (PCR). Deficient mismatch repair/MSI-H has been shown to be a predictive biomarker for the efficacy of immunotherapy, has been reported in approximately 2–3% of CCA, and may be found in both iCCA and eCCA with unclear distribution [81,82,83,84]. While data is limited with regard to prognostic implications, some evidence may suggest that dMMR/MSI-H status is a beneficial prognostic marker in CCA [85].

### 6.2. High Microsatellite Instability Targeted Therapy

#### 6.2.1. Pembrolizumab

Pembrolizumab was evaluated for use in non-colorectal, MSI-H/dMMR, advanced solid malignancies with progression on prior therapy in the phase 2 KEYNOTE-158 study [86]. This study enrolled 233 patients, including 22 patients with CCA. Among all patients, the ORR was 34.3% with an mPFS of 4.1 months (95% CI 2.4–4.9) and mOS of 23.5 months (95% CI 13.5-not reached). TRAEs were observed in 64.8% of patients, with the most common including fatigue (14.6%), pruritis (12.9%), diarrhea (12.0%), and asthenia (10.7%). Grade 3–4 TRAEs were observed in 14.6%, with the most common including pneumonitis, severe skin reactions (1.3% each), fatigue, colitis, and hepatitis (0.9% each). The updated analysis reported an ORR of 30.8% (95% CI 25.8–36.2), with mPFS of 3.5 months (95% CI 2.3–4.2) and mOS of 20.1 months (95% CI 14.1–27.1) [87]. As a result, pembrolizumab received FDA approval for the treatment of MSI-H/dMMR solid tumors with progression on prior therapy, including CCA.

#### 6.2.2. Dostarlimab

Similarly, dostarlimab, an anti-PD1 antibody, has been evaluated in the phase 1 GARNET trial, which included 144 patients with dMMR or POLE mutated, non-endometrial solid tumors with progression on prior therapy [88]. This study only included one patient with a biliary neoplasm but did demonstrate an ORR of 38.7% (95% CI 29.4–48.6) in patients with dMMR tumors. As such, this may be a potential option for dMMR/MSI-H CCA.

## 7. High Tumor Mutation Burden

### 7.1. High Tumor Mutation Burden Pathophysiology

Tumor mutation burden is the quantification of genetic mutations within a cell. This may be evaluated using next-generation sequencing of tumor DNA, and the presence of ≥10 mutations/megabase (mut/Mb) is frequently referred to as high tumor mutation burden (TMB-H) [89]. A high tumor mutation burden may be seen in conjunction with dMMR/MSI-H, although not exclusively, and may be predictive of responsiveness to immunotherapy [89]. In the case of CCA, different thresholds for defining TMB-H have been used, with one study reporting an incidence of 3.8% in iCCA using a TMB cutoff of 10 mut/mB [90], while another, using a cutoff of 17 mut/mB, found TMB-H in 3.5% of iCCA and 2% of eCCA [91]. There is conflicting data regarding the prognostic value of TMB-H status in iCCA [92,93]. This discrepancy may result from the varying frequency of immune checkpoint inhibitor use in these studies. Actionable targets in CCA are summarized in Table 2.

### 7.2. High Tumor Mutation Burden Targeted Therapy

A high tumor mutation burden is also a potential target in CCA. The previously mentioned KEYNOTE-158 trial was subsequently evaluated to determine the efficacy of pembrolizumab in patients with TMB-H advanced solid malignancies [94]. An ORR of 29% was observed in 102 patients with TMB-H tumors, whereas an ORR of 6% was observed in 688 patients without TMB-H tumors. Based on the results of this trial, the FDA granted approval of pembrolizumab for the treatment of non-colorectal, advanced, TMB-H, solid malignancies. It is important to note that this trial did not include any patients with biliary tract malignancies in the TMB-H group.

**Figure 2:**
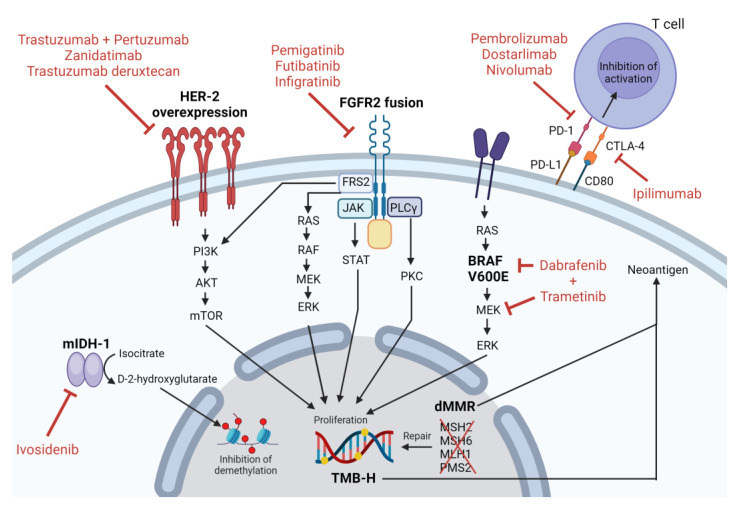
Depiction of targets of interest in cholangiocarcinoma. Note: Image created using biorender.com.

CheckMate 848 was a phase 2 trial that enrolled 212 patients with advanced TMB-H (by tissue biopsy or blood-based assay) solid malignancies who were immunotherapy naïve and had progressed on prior therapy [95]. Patients were randomized 2:1 to receive ipilimumab plus nivolumab versus nivolumab monotherapy. Those receiving ipilimumab plus nivolumab who had TMB-H status determined via tissue assay demonstrated an ORR of 35.3% (95% CI 24.1–47.8) with an mPFS of 4.1 months (95% CI 2.8–11.3) and mOS of 14.5 months (95% CI 7.7-not evaluable). Based on the results of this study, ipilimumab plus nivolumab may be considered in patients with TMB-H CCA. Pertinent clinical trials in CCA are listed in Table 3. A depiction of the various targets, as well as the therapeutics directed at these targets, is shown in Figure 2.

## 8. Other Uncommon/Potential Novel Targets in Cholangiocarcinoma

### 8.1. NTRK

Neurotrophic tyrosine receptor kinase (*NTRK*) is a family of three genes (*NTRK1*, *NTRK2*, and *NTRK3*) that encode tropomyosin receptor kinases (TRKA, TRKB, and TRKC) that play a role in neuronal development [96,97]. Binding of neurotrophins triggers TRK activation, leading to downstream signaling through phospholipase C, mitogen-activated protein kinase (MAPK), or phosphatidylinositol 3 kinase (PI3K) pathways [96,97]. *NTRK* fusion, similar in mechanism to *FGFR2* fusion, stems from chromosomal rearrangement, resulting in the replacement of the 3′ region of *NTRK* with a binding partner that allows for dimerization of TRK and activation of signaling in the absence of neurotrophins [97]. *NTRK* fusion may be observed in <1% of solid malignancies, and has been reported in <1% of cases of CCA [96,98,99].

Entrectinib is an inhibitor of TRKA, TRKB, TRKC, ROS1, and ALK [100]. One study reported the combined results of two phase 1 trials, ALKA-372-001 and STARTRK-1, performing a subgroup analysis of patients with solid tumors having gene fusions involving *NTRK1/2/3*, ROS1, or ALK who were treated with the recommended phase 2 dose of entrectinib and had not received prior treatment with a tyrosine kinase inhibitor [101]. Thirty patients were included in this analysis, of which 25 were evaluable. Of these, three patients had *NTRK1/2/3* gene fusion (none with CCA), and all three manifested a response, suggesting promise in using entrectinib in the management of solid malignancies with *NTRK1/2/3* fusion. Among all 119 phase 1 patients, the most common TRAEs included fatigue (46%), dysgeusia (42%), paresthesia (29%), nausea (28%), and myalgia (23%). Grade 3 TRAEs included fatigue (4%), weight increase (2%), diarrhea (1%), and arthralgia (1%). A subsequent analysis was conducted, including data from three phase 1/2 trials (ALKA-372, STARTRK-1, and STARTRK-2), and included patients with *NTRK* fusion-positive, advanced solid malignancies who were tyrosine kinase inhibitor naïve and received entrectinib 600 mg daily [102]. Fifty-four patients were included in the final analysis, including 1 with CCA, with an ORR of 31% and a median duration of response of 10 months.

Larotrectinib is a highly selective, small-molecule TRK inhibitor that was evaluated in a combined analysis of a phase 1, a phase 1–2, and a phase 2 study, including children and adults with advanced solid malignancies harboring a TRK fusion [103]. This analysis included 55 patients, including 2 patients with CCA, and ultimately demonstrated an ORR of 75% (95% CI 61–85), with 55% of patients remaining progression-free at 1 year. The most common TRAEs included increased AST or ALT (38%), dizziness (25%), fatigue, nausea, constipation (16% each), increased body weight, and vomiting (11% each). The most common grade 3 TRAEs included increased AST or ALT (5%), dizziness, nausea, anemia, and a decreased neutrophil count (2% each).

Based on the results of these studies, *NTRK* fusion-targeted agents may have benefit in this small subpopulation of patients with CCA.

### 8.2. RET

*RET* proto-oncogene (*RET*) encodes a receptor tyrosine kinase, and fusion of the 3′ terminal of *RET* to the 5′ domain of RET finger protein has been implicated in oncogenesis [104]. RET fusion has been observed in thyroid, lung, colorectal, breast, and salivary gland cancers, and this mutation is rare in CCA [105]. 

Pralsetinib is an inhibitor of RET kinases, including fusion proteins stemming from *RET* fusion [106]. ARROW was a phase 1/2 trial enrolling 29 patients with *RET*-altered solid malignancies, including 3 patients with CCA [106]. This study demonstrated an ORR of 55% (95% CI 35–77) with mPFS of 7 months and mOS of 14 months. The most common TRAEs included increased AST (38%), increased ALT (34%), neutropenia (34%), anemia (31%), constipation (24%), decreased white blood cell count (21%), thrombocytopenia, hypertension, and asthenia (17% each). The most common grade 3 TRAEs included neutropenia (31%), anemia (14%), and increased AST (10%).

Selpercatinib is a selective RET kinase inhibitor that was evaluated in LIBRETTO-001, which is an ongoing phase 1/2 basket trial evaluating selpercatinib in patients with *RET*-fusion-positive advanced solid malignancies [107]. The interim analysis included 45 patients, including 2 with CCA, and of 41 efficacy-evaluable patients, the ORR was 43.9% (95% CI 28.5–60.3) with an mPFS of 13.2 months and an mOS of 18.0 months. The most common grade 1-2 TRAEs included dry mouth (29%), ALT increase, AST increase (18% each), diarrhea, QT prolongation, and thrombocytopenia (11% each). The most common grade 3 TRAEs included an ALT increase (16%), hypertension (13%), and an AST increase (11%).

These studies suggest a role for RET inhibition in patients with CCA with RET fusion, although the rarity of this mutation limits evaluation in this population.

## 9. Conclusions

As our understanding of oncogenesis in CCA continues to evolve, multiple targets for intervention have been identified, allowing for the development of targeted, novel therapeutics. Some of these, such as *IDH-1* mutation, HER2 amplification/overexpression, or *FGFR2* fusion/rearrangement, are more common, while others, including *BRAF V600E*, MSI-H, or TMB-H, are less common. These discoveries have paved the way for the development and clinical testing of targeted therapies aimed at these specific genetic anomalies. Multiple agents have been developed and have received FDA approval for use in the second-line setting, with ongoing phase 3 trials evaluating these agents in the first-line setting, although low enrollment has been a barrier in some instances.

Despitethis evolving treatment paradigm, limitations regarding the use of targeted therapies in CCA remain. Reviewing current trial data, most targeted therapies result in, at best, a few months of PFS improvement, with modest improvement in OS. This suggests a need to better understand the resistance mechanisms that develop. For example, in the case of FGFR2 inhibitors, multiple resistance mechanisms have been identified, including mutation of the receptor or activation of feedback survival loops, impairing the duration of response [108,109]. In the case of mIDH-1, previously identified resistance mechanisms include RTK pathway mutations, the development of secondary *IDH* mutations, isoform switching, mitochondrial metabolism, and clonal selection [110]. Future studies must evaluate methods to evade or limit the development of drug resistance.

Additionally, future trials should aim to assess targeted agents in combination with immunotherapy and/or chemotherapy. For example, in the case of HER2 overexpressing biliary tract cancer, a planned trial in South Korea will evaluate the combination of trastuzumab with gemcitabine, cisplatin, and nivolumab (NCT05749900). However, historically, trials evaluating targeted therapies in advanced biliary tract cancer have struggled with patient accrual. In addition to new combinations, new novel therapies are also in development, and continued improvement in the way we inhibit these mechanisms will be important going forward [111].

In summary, targeted therapies offer a new avenue of hope for patients with advanced CCA, though continued research is required to optimize these strategies, overcome resistance mechanisms, and identify the patient populations most likely to benefit. The aim of this article is to outline the current landscape and trial data evaluating targeted therapies in CCA and provide a foundational basis from which future drug optimization and development may grow.

## Figures and Tables

**Table 1:** Select trials evaluating systemic therapy in the first- and second-line settings for cholangiocarcinoma.

Trial Name	Study Arm	Control Arm	Trial Phase	Line of Therapy	Patient Number	Primary EndPoint(s)	ORR	mPFS (Months)	HR, 95% CI (mPFS)	p (mPFS)	mOS (Months)	HR, 95% CI (mOS)	p (mOS)
TOPAZ-1 [12]	Gemcitabine + cisplatin + durvalumab	Gemcitabine + cisplatin	3	First	685	OS	26.7%	7.2 vs. 5.7	0.75, 0.64–0.89	0.001	12.8 vs. 11.5	0.80, 0.66–0.97	0.021
KEYNOTE-966 [13]	Gemcitabine + cisplatin + pembrolizumab	Gemcitabine + cisplatin	3	First	1069	OS	29%	6.5 vs. 5.6	0.86, 0.75–1.00	0.023	12.7 vs. 10.9	0.83, 0.72–0.95	0.0034
ABC-06 [14]	FOLFOX	Best supportive care	3	Second	162	OS	5%	4.0 (FOLFOX arm)	NR	NR	6.2 vs. 5.3	0.69, 0.50–0.97	0.031
NIFTY [15,16]	5-FU + leucovorin + liposomal irinotecan	5-FU + leucovorin	2	Second	178	PFS	12.5%	4.2 vs. 1.7	0.61, 0.44–0.86	0.004	8.6 vs. 5.3	0.68, 0.48–0.95	0.02
NALIRICC-AIO-HEP-0116 [17]	5-FU + leucovorin + liposomal irinotecan	5-FU + leucovorin	2	Second	100	PFS	14.3%	2.8 vs. 2.3	NR	NR	6.9 vs. 8.2	NR	NR

Abbreviations: ORR: objective response rate, mPFS: median progression-free survival, mOS: median overall survival, HR: hazard ratio, 95% CI: 95% confidence interval, NR: not reported.

**Table 2:** Actionable targets in cholangiocarcinoma and incidence by tumor location.

	Incidence	
Target	iCCA	eCCA
*IDH-1* mutation [23,24,25]	20–25%	<1%
HER2 overexpression/amplification [35]	5%	17%
*FGFR2* fusion or rearrangement [50]	15%	<1%
*BRAF V600E* [71,72,73,74,75,76]	1–5%	<1%
Deficient mismatch repair/high microsatellite instability [81,82,83]	2–3%	2–3%
High tumor mutation burden [90,91]	3.5%	2%

**Abbreviations**: iCCA: intrahepatic cholangiocarcinoma; eCCA: extrahepatic cholangiocarcinoma.

**Table 3:** Pertinent clinical trials are evaluating targeted therapy options in cholangiocarcinoma.

Target	Trial Name	Study ARM	Control Arm	Trial Phase	Cohort	Patient Number	Primary EndPoint(s)	ORR	mPFS (Months)	HR, 95% CI (mPFS)	p (mPFS)	mOS (MONTHs)	HR, 95% CI (mOS)	p (mOS)
*IDH-1* mutation	ClarIDHy [29,30]	Ivosidenib	Placebo	3	mIDH-1 CCA w/progression on prior therapy	230	PFS	2%	2.7 vs. 1.4	0.37, 0.25-0.54	<0.0001	10.3 vs. 7.5	0.79, 0.56–1.12	0.09
HER2 overexpression/amplification	MyPathway [39]	Pertuzumab plus trastuzumab	N/A	2a	HER2+, previously treated BTC	39	ORR	23%	2.6 (iCCA), 6.8 (eCCA)	N/A	N/A	3.9 (iCCA), 8.0 (eCCA)	N/A	N/A
	HERIZON-BTC-01 [40]	Zanidatimab	N/A	2b	HER2 2+/3+ (Cohort 1), previously treated BTC	80	ORR	41.3%	5.5	N/A	N/A	Not reported	N/A	N/A
	DESTINY-PanTumor02 [41]	Trastuzumab deruxtecan **	N/A	2	HER2+, previously treated solid malignancies	267 (41 w/BTC)	ORR	37.1%(22.0% among all BTC)	Not reported	N/A	N/A	Not reported	N/A	N/A
*FGFR2* fusion/rearrangement	FIGHT-202 [57]	Pemigatinib	N/A	2	*FGFR2* fusion/rearrangement	107	ORR	35.5%	6.9	N/A	N/A	21.1	N/A	N/A
					*FGFR2* mutation	20		0	2.1			6.7		
					No *FGFR2* abnormality	18		0	1.7			4		
	FOENIX-CCA2 [63,64]	Futibatinib	N/A	2	iCCA w/*FGFR2* fusion/rearrangement	103	ORR	41.7%	8.9	N/A	N/A	20.0	N/A	N/A
	Javle et al. [68]	Infigratinib	N/A	2	CCA w/*FGFR2* fusion or other alteration	61	ORR	14.80%	5.8	N/A	N/A	Not reported	N/A	N/A
	Javle et al. [69]		N/A	2	CCA w/*FGFR2* fusion/rearrangement w/progression on gemcitabine	122	ORR	23.10%	7.3	N/A	N/A	12.2	N/A	N/A
*BRAF V600E*	ROAR [78]	Dabrafenib plus trametinib	N/A	2	BTC w/*BRAF V600E*	43	ORR	51%	9	N/A	N/A	14	N/A	N/A
MSI-H	KEYNOTE-158 [86,87]	Pembrolizumab	N/A	2	Advanced non-colorectal dMMR/MSI-H tumors	351 (22 w/CCA)	ORR	30.80%	3.5	N/A	N/A	20.1	N/A	N/A
TMB-H	KEYNOTE-158 (subgroup analysis) * [94]	Pembrolizumab	N/A	2	Advanced non-colorectal TMB-H tumors	102	ORR	29%	2.1	N/A	N/A	11.7	N/A	N/A
					Advanced non-colorectal non- TMB-H tumors	688		6%	2.1	N/A	N/A	12.8	N/A	N/A
	CheckMate 848 [95]	Ipilimumab plus nivolumab	N/A	2	Advanced, TMB-H solid tumors	148	ORR	22.5-35.3%	2.8-4.1	N/A	N/A	8.5-14.5	N/A	N/A

**Abbreviations**: ORR: objective response rate, mPFS: median progression-free survival, mOS: median overall survival, HR: hazard ratio, 95% CI: 95% confidence interval, BTC: biliary tract cancer, N/A: not available. * Did not include any patients with cholangiocarcinoma in the TMB-H cohort. ** Currently lacking FDA approval for this indication, although evidence would support its use.

## Data Availability

Not applicable.
